# Screening and functional prediction of differentially expressed circular RNAs in human glioma of different grades

**DOI:** 10.18632/aging.202192

**Published:** 2020-12-11

**Authors:** Xiuchao Geng, Yuhao Zhang, Qiang Li, Wang Xi, Wentao Yu, Liang Shi, Xiaomeng Lin, Shaoguang Sun, Hong Wang

**Affiliations:** 1 Faculty of Integrated Traditional Chinese and Western Medicine, Hebei University of Chinese Medicine, Shijiazhuang 050091, PR China; 2 Hebei Key Laboratory of Chinese Medicine Research on Cardio-cerebrovascular Disease, Hebei University of Chinese Medicine, Shijiazhuang 050091, PR China; 3 School of Clinical Medicine, Hebei University, Baoding 071000, PR China; 4 Department of Neurosurgery, Affiliated Hospital of Hebei University, Baoding 071000, PR China; 5 Faculty of Acupuncture-Moxibustion and Tuina, Hebei University of Chinese Medicine, Shijiazhuang 050200, PR China; 6 Department of Neurosurgery, The Second Hospital of Hebei Medical University, Shijiazhuang 050000, PR China; 7 Endoscope Room, Department of General Surgery, Cangzhou Central Hospital, Cangzhou 061001, PR China; 8 Departments of Breast Surgery, Affiliated Hospital of Hebei University, Baoding 071000, PR China; 9 Department of Biochemistry and Molecular Biology, Key Laboratory of Medical Biotechnology of Hebei Province, Hebei Medical University, Shijiazhuang 050017, PR China

**Keywords:** circRNA, glioma, RNA sequencing, functional prediction

## Abstract

Circular RNAs (circRNAs) have a critical regulatory function in human glioma. However, novel circRNAs related to different pathological grades of glioma and their crucial potential function are worth screening and prediction. CircRNA expression profiling was performed for 6 paired high- and low-grade glioma tissues and 5 adjacent normal brain tissues through next-generation sequencing. Quantitative real-time PCR (qRT-PCR) was conducted to validate circRNA expression. Bioinformatics analysis was performed, and circRNA-miRNA-mRNA networks were constructed. The expression and survival data of miRNAs and target genes were examined by GEPIA, Chinese Glioma Genome Atlas (CGGA), ONCOMINE, and cBioPortal databases. The RNA binding proteins (RBPs), open reading frames (ORFs) and N6-methyladenosine (m^6^A) modifications of the identified circRNAs were also predicted. Through multilevel research screening, 4 circRNAs (hsa_circ_0000915, hsa_circ_0127664, hsa_circ_0008362, and hsa_circ_0001467) were associated with glioma of different pathological grades and could be preferred candidates for subsequent functional analysis. Therefore, circRNAs are associated with the different pathological grades of glioma and reveal their potential critical regulatory function. CircRNAs might provide vital molecular biomarkers and potential therapeutic targets for glioma.

## INTRODUCTION

As a commonly seen human intracranial tumor, glioma can be further classified into high-grade (WHO III-IV) and low-grade (WHO I-II) tumors based on histopathological evaluation conducted by the WHO [1]. High-grade glioma (HGG), particularly glioblastoma (GBM) (grade IV), is the most lethal primary brain tumor because of its highly infiltrative growth characteristics. Low-grade gliomas (LGGs), such as diffuse astrocytic and oligodendroglial tumors, have low-grade malignancy and slow growth rates, resulting in a long survival time for patients. There are also several reports of progression of some LGG into HGG in the clinic [2]. However, which factors influence glioma grade is unclear, and the mechanisms underlying the progression, etiology, pathogenesis and molecular characteristics of the different grades of glioma require further study.

As a novel kind of endogenous noncoding RNA (ncRNA), circular RNAs (circRNAs) possess a covalently closed structure with the absence of a 5′ and 3′ end [3]. CircRNAs are specifically expressed in cells or tissues, evolutionarily conserved across species, and relatively stable as a result of resistance to RNase R [4]. CircRNAs have been associated with how malignant tumors are regulated, including gliomas, as indicated by the latest studies. CircRNAs have crucial functions in regulating the cell cycle, apoptosis, angiogenesis, proliferation, invasion, migration, metastasis and radio- and chemoresistance in glioma cells [5–7]. Recent reports have claimed that certain circRNAs are closely related to the pathological grade of gliomas. For example, circFOXO3 [8], circNT5E [9], and hsa_circ_0001730 [10] are differentially expressed in HGG and LGG. CircRNA expression is disordered in HGG compared with matching normal tissues or cells [11]. Currently, as a detection method, next-generation sequencing is a crucial method to explore novel circRNAs. However, the differentially expressed circRNAs of different grades of human glioma have not been fully investigated. Novel circRNAs related to different pathological grades of glioma and their specific crucial functions are worth screening and prediction.

The biological function of circRNAs is mainly reflected in the regulation of gene expression at the transcriptional or posttranscriptional level. Regarding their regulatory mechanisms, circRNAs have been confirmed to act as miRNA sponges to regulate the expression of miRNA target genes by competitively binding miRNA response elements (MREs). Acting as a miRNA sponge or as a competing endogenous RNA (ceRNA) is still one of the most recognized mechanisms. CircRNAs could compete with mRNAs, lncRNAs, and pseudogenes that also contain MREs to adsorb miRNAs, relieve the negative regulation of miRNAs on target gene expression, and improve the function and expression levels of target genes. For instance, circCCDC9 could act as a miR-6792-3p sponge to relieve the negative regulation of miR-6792-3p on its target gene *CAV1* and then inhibit the progression of gastric cancer [12]. CircMAPK4 could function as an oncogene and suppress apoptosis in glioma by sponging miR-125a-3p [13]. Therefore, to explore more circRNAs closely related to multiple cancers, including glioma, the mechanism of miRNA sponges is still a direction worthy of attention and research.

Apart from their function as miRNA sponges, circRNAs can also function as RBP sponges or protein scaffolds and can be translated into either peptides or proteins [14]. According to one report, an internal ribosome entry site (IRES)-driven open reading frame (ORF) is present in circ-SHPRH, which has been reported to be translated into SHPRH-146aa [15]. This finding suggests that the potential translation function may be an important research direction for circRNAs. N6-methyladenine (m^6^A) modification is the most abundant form of posttranscriptional RNA modification in eukaryotes. In recent years, the role of m^6^A in cancers including gliomas has been gradually recognized [16, 17]. It has been reported that N6-methyladenosine (m^6^A) modification could also initiate the translation of circRNAs [18]. In linear mRNAs, m^6^A is usually located around the start and stop codons, and it also appears to be enriched in the junction sequences of circRNAs [19]. Recently, it has been newly reported that m^6^A can also mediate the biogenesis of coding circRNAs [20], and circRNA can not only be modified by m^6^A but also regulate the process of m^6^A modification by binding to the enzyme modified by m^6^A [21]. However, these potential vital functions of circRNAs have not been fully investigated and clarified. Hence, the prediction and analysis of RBP sponges and m^6^A modification of circRNAs requires deep future research.

In this research, we innovatively performed RNA-seq analysis of gliomas of different grades and analyzed and predicted the multiple crucial functions of the identified circRNAs. Our results demonstrated that 4 differentially expressed circRNAs are associated with the pathological characteristics of glioma, and their multilevel functions merit systematic analysis. The potential utility of circRNAs as vital molecular biomarkers or potential therapeutic targets in glioma has been indicated.

## RESULTS

### CircRNA expression profiles

**Figure 1:**
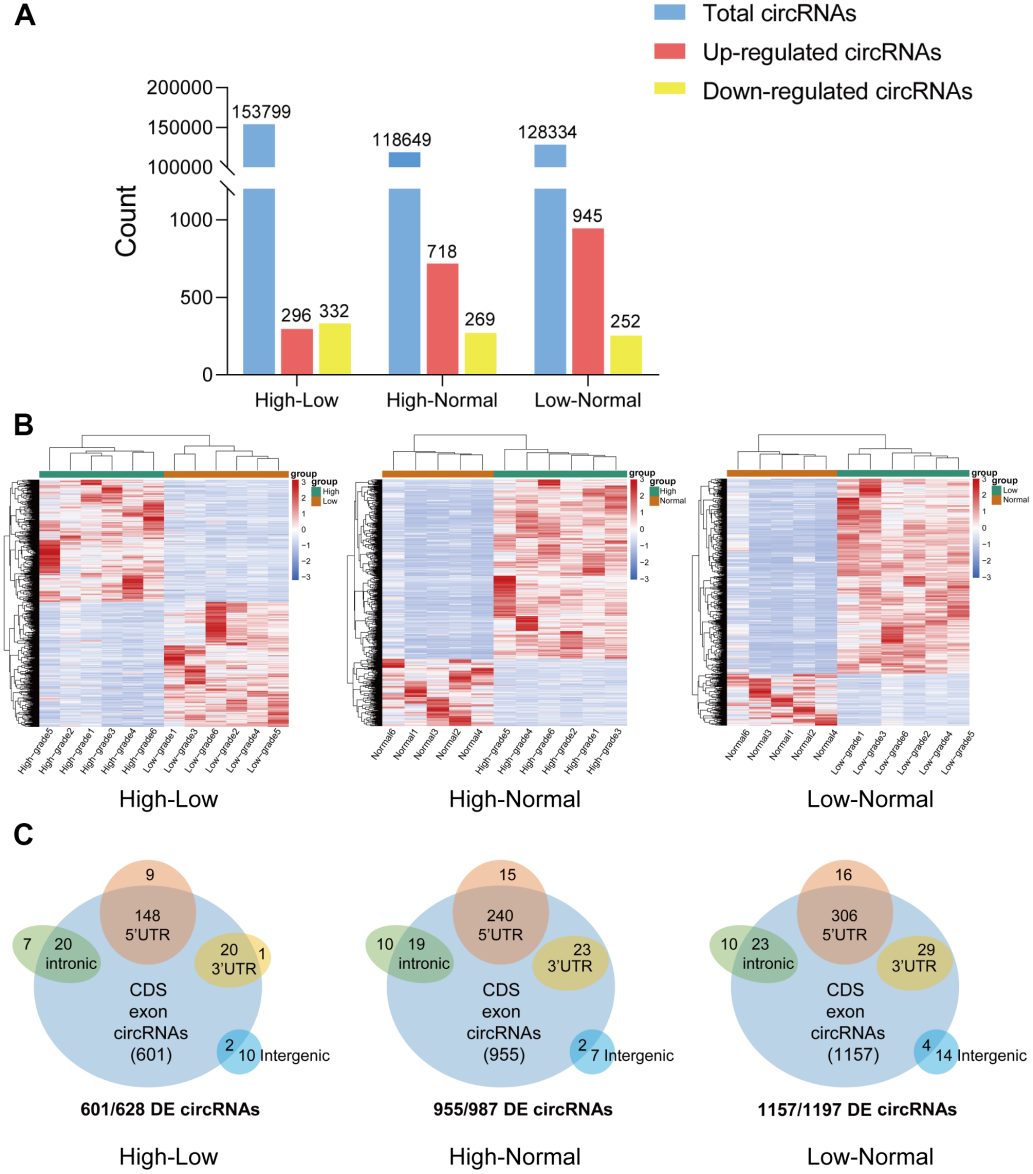
**Overview of circRNA-seq profiles.** (**A**) The quantity of total circRNAs and circRNAs with differential expression (upregulated and downregulated). (**B**) Hierarchical clustering heat map of differentially expressed circRNAs in the three groups. (**C**) CircRNAs with significant differential expression were classified by different genomic loci (exonic, intronic, antisense, intragenic, and intergenic).

**Figure 2:**
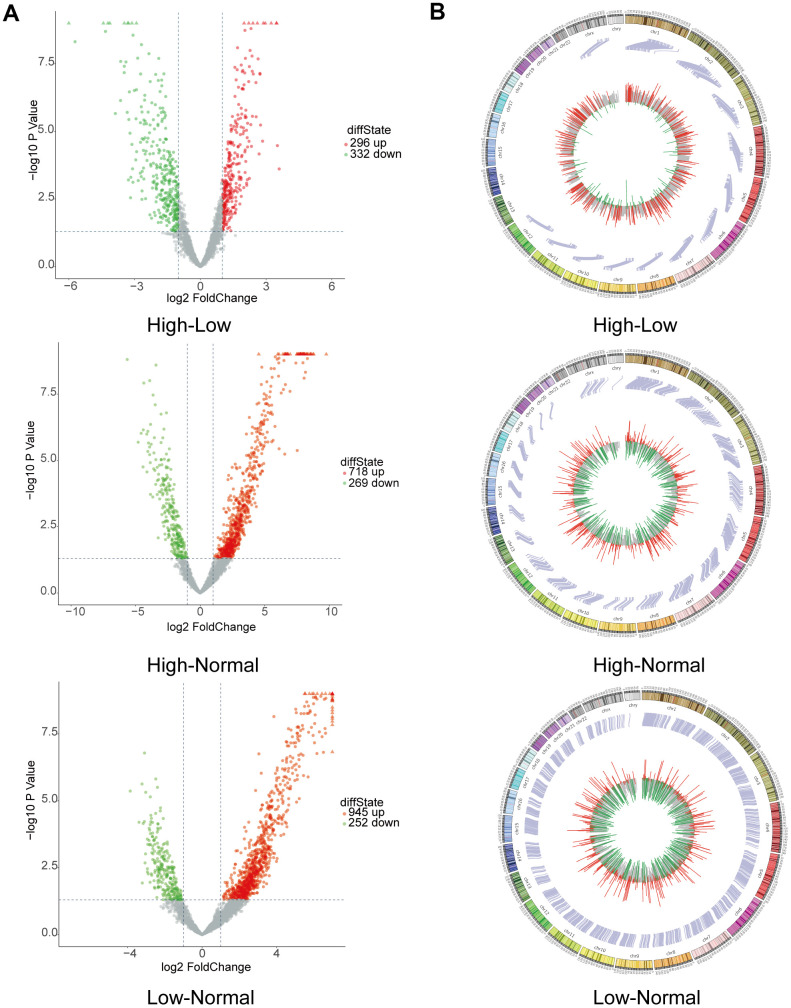
**Bioinformatics analysis by RNA sequencing of circRNAs with differential expression.** (**A**) Volcano plot depicting circRNAs. The volcano plot presents the expression profiles of each of the two groups. The vertical lines indicate a 2-fold (log2 scaled) increase and decrease in expression. The horizontal line indicates a p-value of 0.05 (−log10 scaled). CircRNAs showing significant upregulation are represented by red points. CircRNAs exhibiting significant downregulation are indicated by green points in the plot. (**B**) Circos plot revealing where each circRNA is located on human chromosomes.

CircRNA expression profiles were performed on 6 HGG and 6 LGG patient tissues as well as 5 adjacent normal tissues. After the raw data were quantile normalized, the circRNA expression profiles were observed. A fold change (FC) > 2 and p < 0.05 were the cutoff criteria to identify differentially expressed circRNAs between each pair of groups. Eventually, a total of 153799 circRNAs were discovered in the high-low grade group. Among them, 296 exhibited upregulation and 332 showed downregulation. With regard to the high-normal grade group, 118649 circRNAs were found. Among them, 718 showed upregulation, and 269 exhibited downregulation. For the low-normal grade group, 128334 circRNAs were discovered, among which 945 showed upregulation and 252 exhibited downregulation (Figure 1A). As demonstrated by the hierarchical clustering heatmap (Figure 1B), a distinctive mode of circRNA expression was observed among the three groups. Based on how the different circRNA types were distributed, a large proportion of the circRNAs originate from exons. In comparison, other circRNAs stem from intronic and/or intergenic genomic regions (Figure 1C). How the expression of circRNAs was differentiated to a significant extent between each group is presented in the form of volcano plots (Figure 2A). The Circos plots show all the differentially expressed circRNAs compared with HGG, LGG tissue and adjacent normal brain tissue (Figure 2B), which led to the finding that every single chromosome showed differences in the expression of circRNAs.

### Validation of the circRNA-seq data via qRT-PCR

To verify the circRNA-seq analysis data, we focused on the downregulated circRNAs with certain circBase IDs. Five circRNAs were first selected as examples in further analysis from the most downregulated circRNAs. A design was developed for the divergent primers spanning the back-splice junction sites. The qRT-PCR-amplified products of each candidate circRNA were identified by Sanger sequencing to validate the sequence of head-to-tail splice junctions. The primer sequences are presented in Supplementary Table 1.

**Figure 3:**
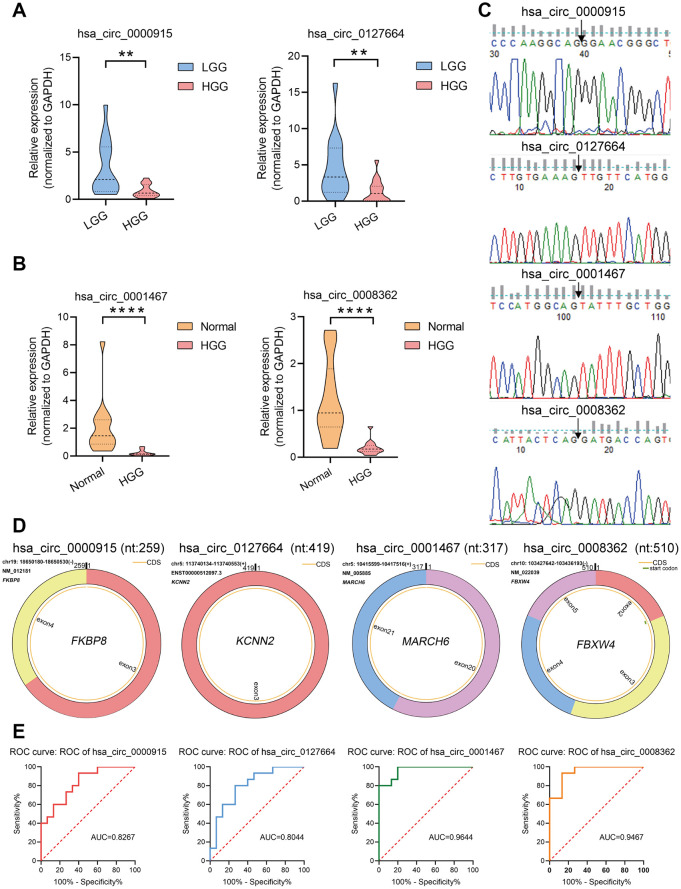
**Verification of circRNA expression using qRT-PCR.** (**A, B**) To confirm the downregulation of four circRNAs observed in circRNA-seq data, hsa_circ_0000915 and hsa_circ_0127664 were found to be downregulated in samples collected from 15 HGG and 15 LGG tissues, and hsa_circ_0001467 and hsa_circ_0008362 were found to be downregulated in 15 HGG tissues and 15 adjacent normal tissues via qRT-PCR. The data are presented as the means ± S.D. of a minimum of 3 independent experiments. *p < 0.05; **p < 0.01; ****p < 0.0001. (**C**) Head-to-tail splice junctions of 4 circRNAs confirmed by Sanger sequencing. (**D**) Characterization information for 4 candidate circRNAs. (**E**) ROC curve analysis of four candidate circRNAs. hsa_circ_0000915 and hsa_circ_0127664 in HGG patients were analyzed against low-grade glioma patients via ROC curves, and hsa_circ_0008362 and hsa_circ_0001467 in HGG were analyzed against normal tissue using ROC curves.

Samples were obtained from 15 HGG tissues, 15 LGG tissues, and 15 adjacent normal tissues for qRT-PCR analysis. The analysis revealed that 4 circRNAs were significantly downregulated, showing the same trend as the RNA-seq results, except 1 circRNA (hsa_circ_0000199) was not significantly differentially expressed. In conclusion, the qRT-PCR results suggested that the RNA-seq was reliable and that most of the screened circRNAs merit further investigation. As shown in Figure 3A, the expression of hsa_circ_0000915 and hsa_circ_0127664 was downregulated in the HGG group compared with the LGG group, and the expression of hsa_circ_0001467 and hsa_circ_0008362 was downregulated in the HGG group compared with the normal group (Figure 3B). Sanger sequencing was used to validate the head-to-tail splice junctions of the above 4 circRNAs (Figure 3C). Through characterization of the identified circRNAs, we found that hsa_circ_0000915 (position: chr19: 18650181-18650530) is encoded from exons 3 and 4 of *FKBP8*. Its splicing length is 259 bp. Moreover, the characterization information for hsa_circ_0000915, hsa_circ_0127664, hsa_circ_0008362, and hsa_circ_0001467 were drawn via circPrimer software [22] (Figure 3D).

### Diagnostic accuracy analysis of candidate circRNAs

To explore the potential diagnostic value of the identified circRNAs, receiver operating characteristic (ROC) curves were analyzed. Relative to low grade, HGG patients showed a downregulation of hsa_circ_0000915 and hsa_circ_0127664 expression. To obtain the diagnostic value of these 2 circRNAs in HGG and LGG patients, a ROC curve analysis was conducted. The AUC was 0.8267 (95% CI: 0.6819–0.9714, p < 0.01) for hsa_circ_0000915 and 0.8044 (95% CI: 0.6457–0.9632, p < 0.001) for hsa_circ_0127664. Moreover, the diagnostic value of hsa_circ_0008362 and hsa_circ_0001467 was also evaluated by ROC curve analysis in HGG and normal tissue. The AUCs for hsa_circ_0008362 and hsa_circ_0001467 were 0.9467 (95% CI: 0.8728–1.000, p < 0.0001) and 0.9644 (95% CI: 0.9088–1.000, p < 0.0001), respectively. The potential diagnostic value of these circRNAs in glioma patients is displayed in Figure 3E.

### GO enrichment and Reactome pathway analysis

**Figure 4:**
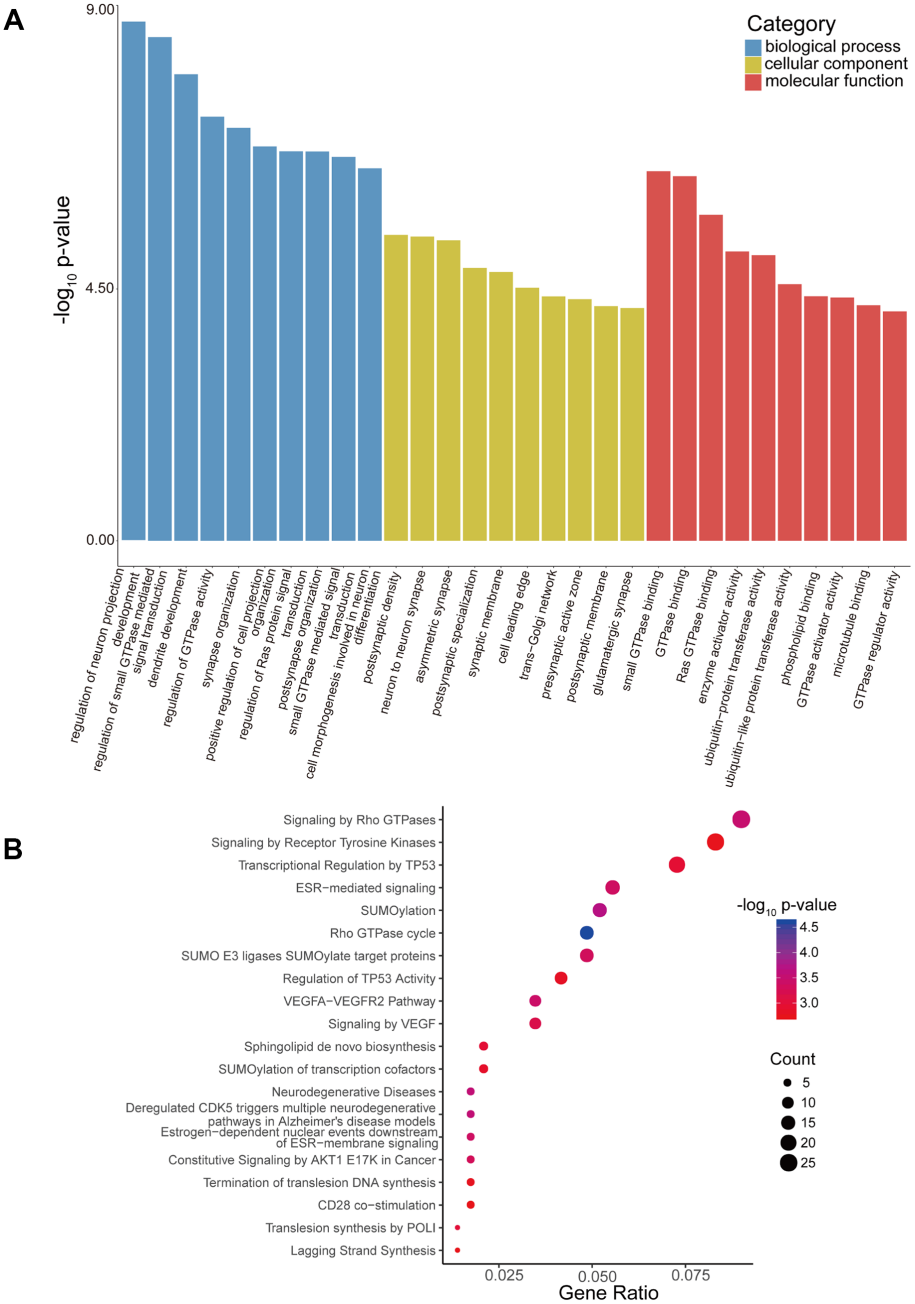
**GO enrichment and Reactome pathway analyses of host genes of differentially expressed circRNAs.** (**A**) GO enrichment analysis of host genes for circRNAs with differential expression. The ten most enriched GO terms in the MF, BP, and CC categories for the host genes. (**B**) Bubble map of Reactome pathway analysis of the top 20 predominant pathways. The X-axis denotes the enriched differential gene ratio in each pathway. The Y-axis represents the name of the significantly enriched pathway. The p-values are expressed by variations from red to blue. A deeper blue color indicates a greater significant difference.

By conducting Gene Ontology (GO) enrichment analysis in the biological process (BP), cellular component (CC), and molecular function (MF) categories, the host genes (linear counterparts) of the differentially regulated circRNAs in high-low grade were investigated, based on which their potential roles were deduced. The 10 most enriched BP, CC and MF GO terms of dysregulated circRNAs from the high-low grade glioma group are shown in Figure 4A. The regulation of neuron projection development, GTPase binding, and postsynaptic density were the most enriched GO entries in the BP, MF, and CC categories, respectively. Then, we performed Reactome pathway analysis, and 20 ranking predominant pathways are displayed in Figure 4B. According to the Reactome analysis, the top three enriched and meaningful pathways were signaling by Rho GTPases (Reactome, R-HSA-194315; p= 0.000312945), signaling by receptor tyrosine kinases (Reactome, R-HSA-9006934; p= 0.001761105) and transcriptional regulation by *TP53* (Reactome, R-HSA-3700989; p= 0.001084954).

**Figure 5:**
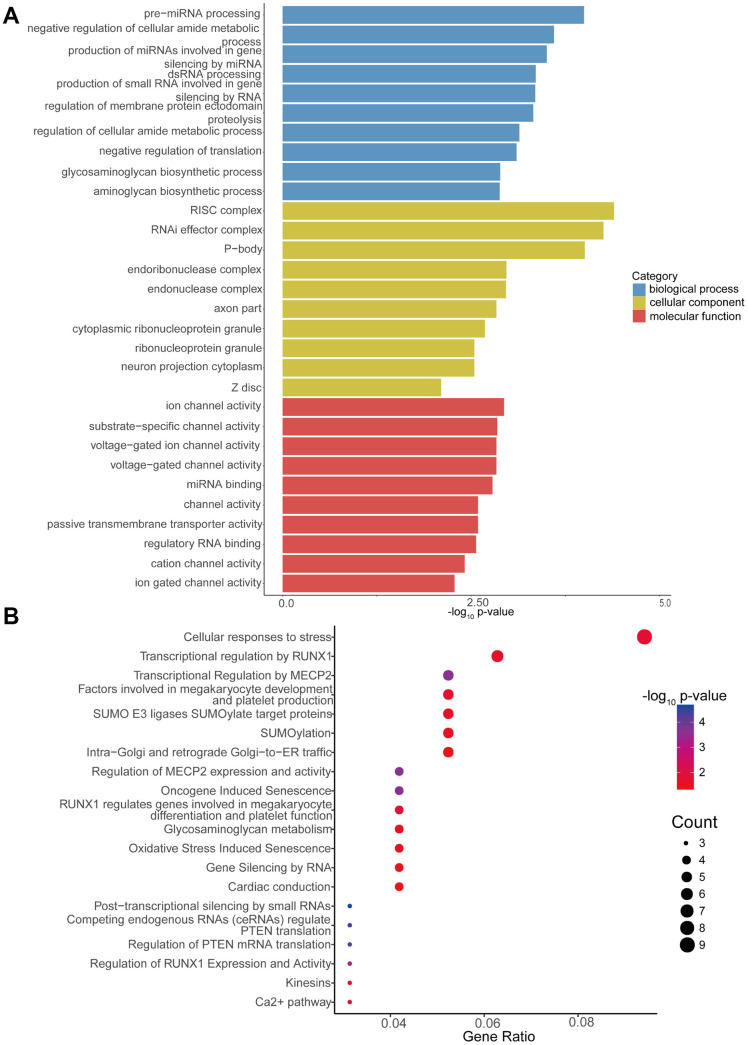
**GO enrichment and Reactome pathway analyses of target genes of differentially expressed circRNAs.** (**A**) GO enrichment analysis of target genes for circRNAs with differential expression. The 10 most significantly enriched GO terms in the MF, BP, and CC for the target genes of all miRNAs with differential expression. (**B**) Bubble map of Reactome pathway analysis of the top 20 predominant pathways. The X-axis denotes the enriched differential gene ratio in each pathway. The Y-axis represents the name of the significantly enriched pathway. The p-values are expressed as variations from red to blue. A deeper blue color indicates a greater significant difference.

Additionally, GO enrichment and Reactome pathway analysis of the downstream target genes in high-low grade was conducted. Figure 5A presents the 10 most enriched BP, CC and MF GO terms for the target genes in high-low grade. PremiRNA processing, ion channel activity, and RISC complex were the most enriched GO entries in the BP, MF, and CC categories, respectively. In addition, the top 20 predominant pathways were displayed by Reactome pathway analysis (Figure 5B). According to the Reactome analysis, the top three pathways were cellular responses to stress (Reactome, R-HSA-2262752; p= 0.015340627), transcriptional regulation by *RUNX1* (Reactome, R-HSA-8878171; p=0.021079352) and transcriptional regulation by *MECP2* (Reactome, R-HSA-8986944; p=0.000234161).

### CircRNA-miRNA-mRNA network analysis

**Figure 6:**
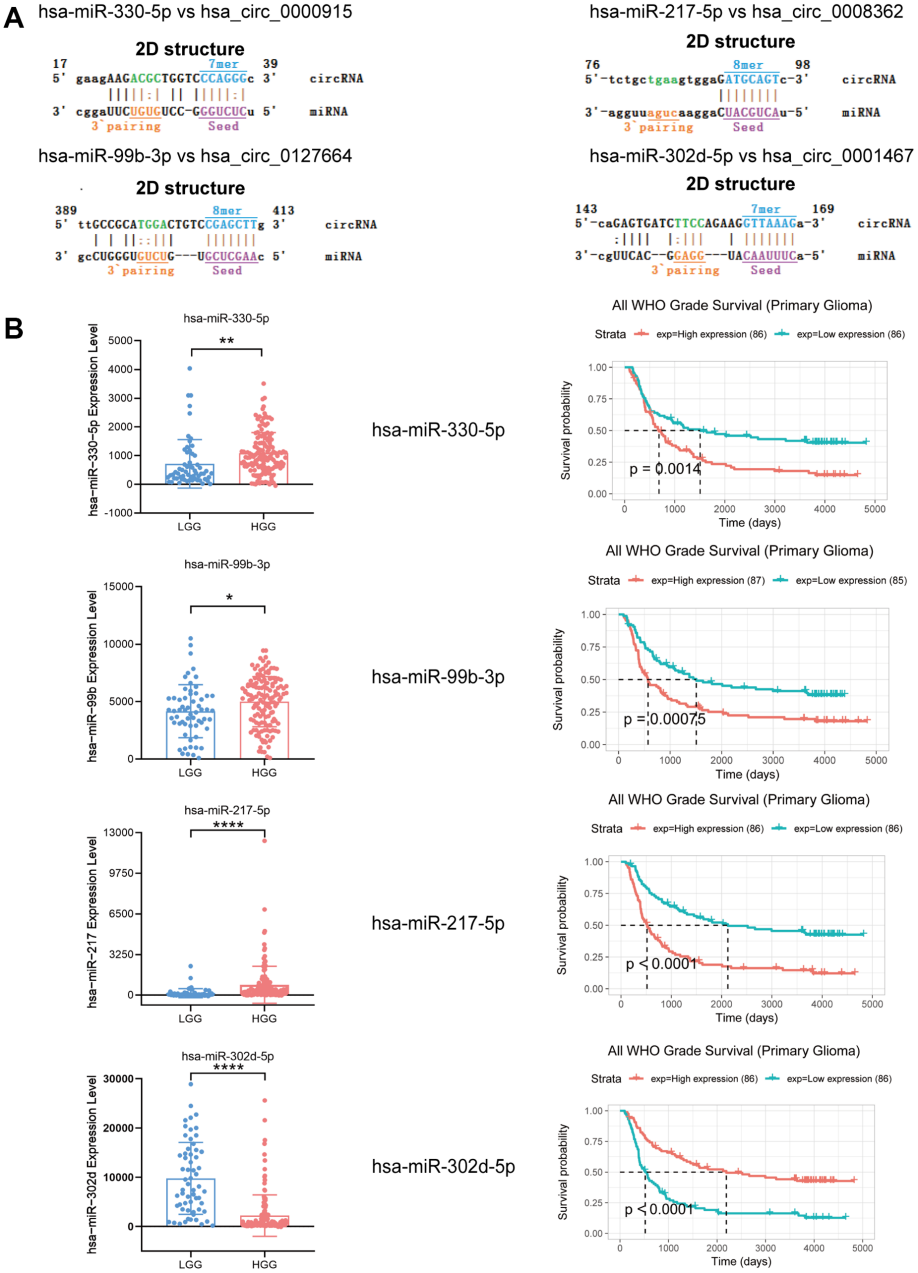
**Expression and survival analysis of 4 target miRNAs and sequence analysis.** (**A**) Sequence analysis results for MREs of identified circRNAs and target miRNAs. (**B**) In comparison to LGG patients, hsa-miR-330-5p, hsa-miR-99b-3p and hsa-miR-217-5p were upregulated in HGG, but hsa-miR-302d-5p was downregulated. Survival analysis of 4 target miRNAs. *p < 0.05; **p < 0.01; ****p < 0.0001.

Emerging studies have revealed that circRNAs could be miRNA sponges, which are effective in regulating how mRNA is expressed [23, 24]. In this paper, miRNAs that could potentially bind circRNAs were predicted using miRanda software (total score ≥ 150, total energy ≤ −20). A total of 129 miRNAs were found to be potential targets of the 4 identified circRNAs (Supplementary Table 2). Based on the bioinformatics analysis and combined with the research reports about miRNAs related to glioma in PubMed, we found that the most likely potential target miRNAs for hsa_circ_0000915 included hsa-miR-6765-3p and hsa-miR-330-5p. The most likely potential target miRNAs for hsa_circ_0127664 included hsa-miR-3945 and hsa-miR-99b-3p. hsa-miR-4656 and hsa-miR-217-5p were also predicted to be potential targets of hsa_circ_0008362. hsa-miR-6827-5p and hsa-miR-302d-5p were predicted to be potential targets of hsa_circ_0001467. The partially screened detailed sequence analysis of MREs for hsa_circ_0000915, hsa_circ_0127664, hsa_circ_0008362 and hsa_circ_0001467 is presented in Figure 6A.

In addition, 4 target miRNAs of 4 identified circRNAs were systematically analyzed using microRNA microarray data provided by the CGGA database (LGG group n = 60; HGG group n = 138), as they were extensively reported as mRNA regulators in gliomas. For the study, hsa-miR-330-5p, hsa-miR-99b-3p, hsa-miR-217-5p, and hsa-miR-302d-5p were taken as target miRNAs of these 4 circRNAs that had been discovered. hsa-miR-330-5p, hsa-miR-99b-3p, and hsa-miR-217-5p expression levels were found to show an increasing trend in the HGG group in comparison to the LGG group, but hsa-miR-302d-5p showed a decreasing trend. As shown by survival analysis, the group with enhanced hsa-miR-330-5p, hsa-miR-99b-3p, and hsa-miR-217-5p expression showed a reduced length of survival, and hsa-miR-302d-5p showed the opposite trend (Figure 6B).

**Figure 7:**
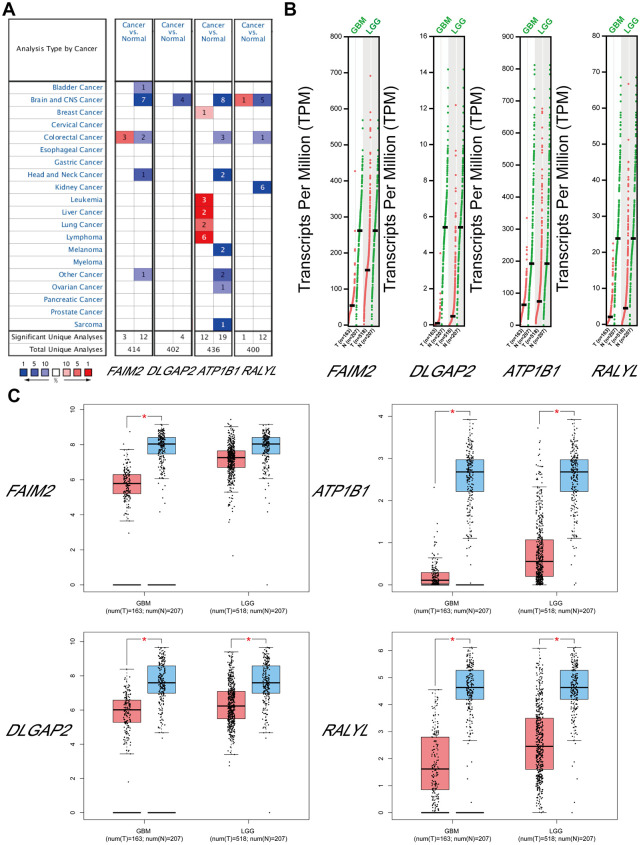
**The expression levels of target mRNAs.** (**A**) The expression levels of *FAIM2*, *DLGAP2*, *ATP1B1* and *RALYL* in multiple cancers using ONCOMINE databases. (**B, C**) The expression levels of *FAIM2*, *DLGAP2*, *ATP1B1* and *RALYL* in GBM and LGG using GEPIA databases.

The top target genes of miRNAs were predicted by TargetScan and miRDB (Criteria: Cumulative weighted context++ score ≤ −0.24 for TargetScan, Target Score ≥ 90 for miRDB). By analysis, we found that *FAIM2*, *DLGAP2*, *ATP1B1,* and *RALYL* could be the targets of hsa-miR-330-5p, hsa-miR-99b-3p, hsa-miR-217-5p and hsa-miR-302d-5p, respectively. We compared the expression levels of *FAIM2*, *DLGAP2*, *ATP1B1* and *RALYL* using ONCOMINE databases and found that these 4 genes were significantly downregulated in brain and CNS cancer compared with the normal group (Figure 7A). The expression data for these target genes in patients with GBM and LGG were examined by GEPIA. Compared with the normal group, *FAIM2*, *DLGAP2*, *ATP1B1* and *RALYL* were downregulated in GBM and LGG patients (Figure 7B, 7C).

The survival analysis was performed with the CGGA database. The group with low *FAIM2*, *DLGAP2*, *ATP1B1* and *RALYL* expression showed a reduced length of survival (Supplementary Figure 1A). We analyzed the *FAIM2*, *DLGAP2*, *ATP1B1* and *RALYL* alterations using the cBioPortal online tool for Glioblastoma Multiforme (TCGA, Firehose Legacy). Four genes were altered in 28 (21%) queried patients/samples (136 samples) (Supplementary Figure 1B). These results indicated that these genes were likely to be glioma-related tumor suppressor genes. The circRNA-miRNA-mRNA network was diagrammed with the assistance of Cytoscape software (Supplementary Figure 2) (Supplementary Table 3).

### RBP binding and m^6^A modification function prediction

**Figure 8:**
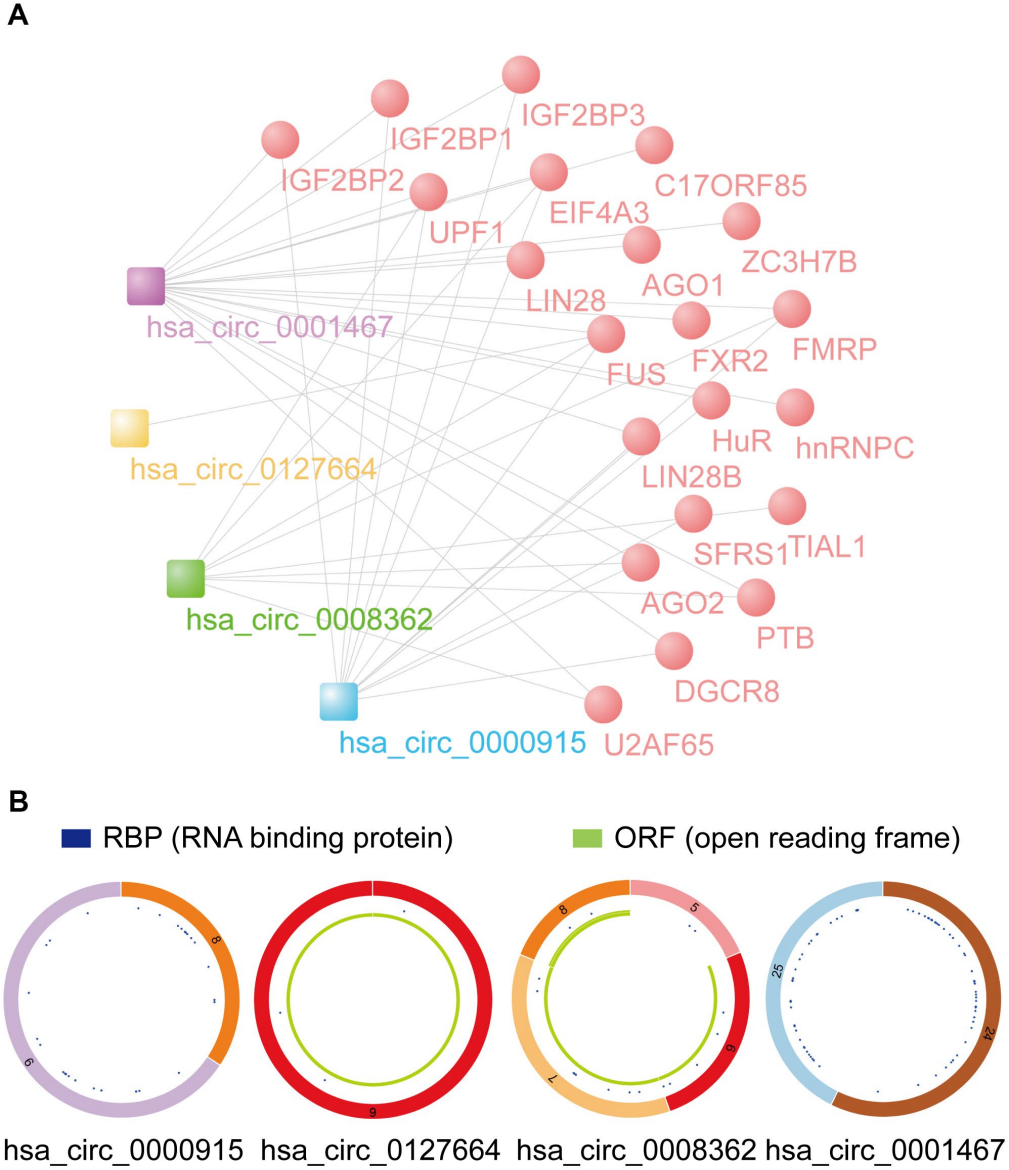
**Prediction of RBP binding and m^6^A modification functions of selected circRNAs.** (**A**) RBP binding to 4 identified circRNAs was predicted by CSCD data. (**B**) The structural patterns of 4 circRNAs analyzed using the CSCD database. All four circRNAs were searchable in the CSCD database. The RBP, and ORF information is shown.

According to the data from the Cancer-Specific CircRNA Database (CSCD), RBPs binding with 4 identified circRNAs were drawn via Cytoscape software (Figure 8A). Twenty-two vital RBPs, IGF2BP1, IGF2BP3, EIF4A3, hnRNPC, and AGO2, can bind to circRNAs and may play important biological functions in glioma. According to the data from CSCD, the structure of 4 circRNAs was determined. Diagrams containing information about RBPs and ORFs are presented in Figure 8B. In addition, an interaction map of the difference in gene expression of RBPs between GBM and LGG and cancer pathway activity (activation and inhibition) was analyzed by GSCALite (Supplementary Figure 3). For example, we found that IGF2BP2, which is closely related to GBM and LGG, can be closely related to the mesenchymal transition (EMT) pathway of cancer progression.

According to data from the circBank database, the m^6^A levels for the 4 circRNAs were obtained and are shown in Table 1. We then analyzed their m^6^A modification sites using the SRAMP predictor tool. Only high confidence m^6^A sites are listed (Table 2). It was found that hsa_circ_0127664 and hsa_circ_0008362 had a relatively high m^6^A modification. The local m^6^A site structure visualization is displayed in Supplementary Figure 4. The prediction of coding proteins of identified circRNAs was analyzed by circRNADb, and hsa_circ_0000915 and hsa_circ_0001467 had IRES as well as ORFs, revealing their potential functions in coding proteins (Table 3).

**Table 1:** The m^6^A levels of circRNAs based on circBank.

**circBase ID**	**circBank ID**	**chr**	**start**	**end**	**strand**	**m^6^A Levels**
hsa_circ_0127664	hsa_circKCNN2_003	chr5	113740134	113740553	+	0.6784
hsa_circ_0008362	hsa_circFBXW4_008	chr10	103427642	103436193	-	0.0507
	hsa_circFBXW4_008	chr10	103432671	103436193	-	0.391
hsa_circ_0000915	hsa_circFKBP8_005	chr19	18650180	18650530	-	0.0521
	hsa_circFKBP8_005	chr19	18650180	18650530	-	0.0005
hsa_circ_0001467	hsa_circMARCH6_047	chr5	10415599	10417516	+	0.0005

**Table 2:** The m^6^A sites of circRNAs based on SRAMP.

**circBase ID**	**Position**	**Sequence context**	**Score (combined)**	**Decision**
hsa_circ_0127664	14	---------UUGUUCAUGGUGGACAAUGGAGCAGAUGACUGGAGA	0.626	m^6^A site (High confidence)
	400	AUUAUGGAUAAUUGCCGCAUGGACUGUCCGAGCUUGUGAAAG---	0.676	m^6^A site (High confidence)
hsa_circ_0008362	357	UCGGCUCAUGAACAGGAGGUGAACUGUGUGGAUUGCAAAGGGGGC	0.637	m^6^A site (High confidence)
	402	AUCAUUGUGAGUGGCUCCAGGGACAGGACGGCCAAGGUGUGGCCU	0.632	m^6^A site (High confidence)
	467	GCAGUGCUUACACACCAUCCAGACUGAAGACCGAGUCUGGUCCAU	0.614	m^6^A site (High confidence)

**Table 3:** The coding potential prediction of the identified circRNAs.

**circRNA ID**	**Gene symbol**	**Genomic length**	**Spliced length**	**Best transcript**	**Start position**	**End position**	**Protein length**
hsa_circ_0000915	FKBP8	350	259	NM_012181	106	2r+154	188 aa
hsa_circ_0001467	MARCH6	1917	317	NM_005885	311	2r+21	114 aa

## DISCUSSION

Circular RNAs (circRNAs) have a critical regulatory function in human glioma [25]. As a detection method, next-generation sequencing is a crucial method to explore novel circRNAs. However, regarding circRNA-seq data for glioma, most previous studies have focused on HGG, particularly glioblastoma (GBM, WHO IV), and thus, dysregulated circRNAs have only been analyzed between glioma and normal tissues [9]. The differentially expressed circRNAs of different grades of human glioma have not been fully investigated. Novel circRNAs related to different pathological grades of glioma and their crucial function are also worth screening and prediction. In this research, we simultaneously investigated the circRNA expression profile of different grades of human glioma. Subsequently, to verify the RNA-seq data, the circRNA expression level in tissues was evaluated by qRT-PCR. To determine the potential clinical diagnostic value of circRNAs with differential expression, ROC curves were analyzed. In addition, to preliminarily explore the multilevel functions as well as potential mechanisms associated with malignant development of glioma, GO enrichment, Reactome pathway, and circRNA-miRNA-mRNA network analyses were performed. The expression and overall survival data for the target miRNAs and mRNAs were analyzed and examined using the GEPIA, CGGA, ONCOMINE, and cBioPortal databases. We screened 4 important circRNAs and predicted their potential functions. As a novel direction worth exploring for circRNAs, m^6^A modification levels, RBPs, and ORFs were also predicted for further analysis.

We found that hundreds of circRNAs, with upregulation and downregulation, were significantly differentially expressed (FC ≥ 2 or ≤ 0.5 and p < 0.05) in different grade glioma patients. As revealed by the RNA-seq analysis carried out in the high-normal and low-normal groups, the number of upregulated circRNAs exceeded that of circRNAs exhibiting downregulation. The same phenomenon was observed in a study published by Xia et al. [26]. However, in the high-low group, we found more downregulated circRNAs, which aroused our interest. In addition, a large proportion of the circRNAs identified in our study originated from exons. In comparison, other circRNAs stemmed from intronic and/or intergenic genomic regions, which was also observed in a study published by Zhu et al. [27].

Despite many studies focusing on upregulated circRNAs in multiple cancer types, other studies have suggested that a majority of circRNAs with active expression play a significant role in normal physiological functions [28]. Relative to colorectal and ovarian cancer tissues, a high proportion of circRNAs are abundant in normal human tissues [29]. Many studies have revealed that certain circRNAs are downregulated in tumors compared with normal tissues and cells [30–33], which indicates that these circRNAs may function as suppressor genes and have a protective function in inhibiting cancer. Hence, we chose specific downregulated circRNAs for further research. Five downregulated circRNAs were first selected from the most downregulated circRNAs, and the RNA-seq reliability was evaluated with qRT-PCR. In 15 paired HGG and LGG tissues and 15 adjacent normal tissues, the expression of these 5 circRNAs was validated via RT-qPCR. Among the 5 circRNAs, four exhibited a clear conformance with RNA-seq, indicating that the RNA-seq data were reliable. Furthermore, we found that the selected representative circRNA, hsa_circ_0001467, was distinctly downregulated in glioma tissues, which is related to its diagnostic value for glioma patients and shows its potentially important clinical significance. Thus, downregulated circRNAs should not be neglected because they may serve as a novel tumor suppressive factor and potential target for new therapies in human cancer as well as other diseases [34–36].

The differential expression of circRNAs is reasonably related to their parental genes because they are mainly encoded from exons and/or introns of host genes [3, 37]. To explore the roles of circRNAs through this potential mechanism, the biological functions and identified predominant pathways of their host gene as well as target genes were conducted through GO enrichment and Reactome pathway analyses. According to GO enrichment analysis, the deregulation of circRNAs was related to the regulation of neuron projection development, GTPase binding, and postsynaptic density. According to the Reactome analysis of host genes, the top three pathways were signaling by Rho GTPases, signaling by receptor tyrosine kinases and transcriptional regulation by TP53. However, the top three pathways were cellular responses to stress, transcriptional regulation by RUNX1, and transcriptional regulation by MECP2 in the Reactome pathway analyses of target genes. We noted that the Rho GTPase pathway has been shown to be associated with infiltrative growth and invasion of glioblastoma [38, 39]. In addition, RUNX1 is considered to a key factor participating in the malignant biological behavior of glioma cells [40]. It is highly expressed and could contributes to the mesenchymal subtype of GBM in a TGFβ pathway-dependent manner [41]. Although many studies have analyzed the host genes of dysregulated circRNAs based on GO enrichment and pathway analyses [27, 42], we support that GO and pathway analyses aimed at target genes can also better predict circRNA function.

Increasing evidence suggests that circRNAs are related to various diseases that humans can develop, particularly carcinomas, such as glioma [10], gastric cancer [43, 44], hepatocellular carcinoma [45–47], pancreatic cancer [48], thyroid carcinoma [49] and breast cancer [50]. Thus, it is speculated that circRNAs might assist with the diagnosis and treatment of these human diseases. Our results also revealed that these circRNAs were suitable and sensitive diagnostic markers in identifying glioma. Additionally, many studies have indicated that circRNA is capable of acting as a miRNA sponge, which leads to interference with miRNAs or other varieties of molecules associated with the target genes to be inhibited [23]. Considering the significance of miRNAs in the pathogenesis of glioma, it was suspected that the identified circRNAs might cause malignant development of glioma through interactions with miRNAs. Therefore, miRNAs targeted by these differentially expressed circRNAs were predicted. For instance, hsa_circ_0000915 has the potential to bind to hsa-miR-330-5p. As demonstrated in a prior study, hsa-miR-330-5p is related to the stemness and development of glioma [11]. In addition, according to our analysis based on the CGGA database, hsa-miR-330-5p also shows important clinical utility. A study on hsa-miR-302d-5p and glioblastoma showed that hsa-miR-302d-5p could function as a tumor inhibitor inhibiting glioblastoma via targeting NF-κB as well as *FGF2* [51]. In addition, miR-217 could target *YWHAG* and promote the viability, proliferation, migration, invasion and mitosis of glioblastoma cells both in vitro and in vivo [52]. Accordingly, the prediction results of circRNAs and miRNAs in this paper deserve further study.

By analysis, we found that *FAIM2*, *DLGAP2*, *ATP1B1* and *RALYL* could be the targets of hsa-miR-330-5p, hsa-miR-99b-3p, hsa-miR-217-5p and hsa-miR-302d-5p, respectively. The data from the GEPIA, CGGA, ONCOMINE, and cBioPortal databases all suggested that these four genes were closely related to glioma and are likely to be tumor suppressor genes.

RBPs can act as activators or inhibitors of circRNA formation and regulate the expression level of circRNAs [53]. Recently, binding to RBPs and the potential coding of circRNAs have attracted the attention of researchers; for example, circ-Amotl1 could bind to PDK1 as well as AKT1, facilitating the cardioprotective nuclear translocation of pAKT [54]. In our research, the potential RBPs of these 4 circRNAs were analyzed. Twenty-two vital RBPs were found, including IGF2BP1, IGF2BP3, EIF4A3, hnRNPC, and AGO2. For instance, it was recently found that E2F1 and EIF4A3 might promote the biogenesis of circSEPT9 and the occurrence and development of triple-negative breast cancer by acting on the miR-637/LIF axis [55]. The interaction of the difference in gene expression of RBPs between GBM and LGG and cancer pathway activity was also analyzed. We found that some RBP genes were closely related to GBM and LGG, and they were predicted to have a close correlation with multiple cancer pathways. In our research, IGF2BP2 was predicted to be closely related to the EMT pathway of cancer progression. A recent study also confirmed this conclusion. It has been reported that miR-138 may function as a tumor inhibitor by directly inhibiting IGF2BP2 and suppressing EMT during the progression of LGG [56]. Hence, RBPs of circRNAs in this paper merit further study.

A large proportion of circRNAs, which are derived from coding genes and have ORFs, show potential functions of coding proteins [20]. In one study, circRNAs showed many m^6^A consensus motifs, and a m^6^A site was enough to initiate circRNA translation in a cap-independent fashion involving the m^6^A reader YTHDF3 and the translation initiation factors eIF4G2 and eIF3A. circRNA translation efficiency can be affected by the level of m^6^A modification [18, 57]. Interestingly, after searching the literature and GEPIA databases, it was found that eIF4G2 and YTHDF3 were upregulated in glioma compared with normal brain tissues [58] (GEPIA2 screening condition: log2foldchange cutoff=1, p-value cutoff=0.01). In another study, m^6^A modification was also discovered to be common in circRNAs [59]. Recently, m^6^A was reported to mediate the biogenesis of coding circRNAs [20] and could modulate cytoplasmic export and promote CRC liver metastasis [60]. The functional crosstalk and their function in tumor regulation between non-coding RNAs (miRNA, lncRNA, and circRNA) and m^6^A have been explored [16, 61]. Hence, the analysis and validation of m^6^A in circRNAs requires further research. We analyzed the m^6^A modification levels of candidate circRNAs. For example, the m^6^A modification level of hsa_circ_0127664 is 0.6784, which was provided by circBank. The coding proteins prediction of hsa_circ_0001467 suggests that it might function as a coding protein. Thus, this modification in circRNAs also merits further analysis.

## CONCLUSIONS

Through next-generation sequencing and bioinformatics analysis, the important dysregulated circRNAs were determined. Our findings indicated that 4 circRNAs (hsa_circ_0000915, hsa_circ_0127664, hsa_circ_0008362, and hsa_circ_0001467) are associated with the pathological characteristics of glioma and revealed critical regulatory functions at multiple levels. The results presented here might provide vital molecular biomarkers and potential therapeutic targets for glioma.

## MATERIALS AND METHODS

### Clinical samples

HGG and LGG patient tissues and adjacent normal tissues were collected from the Second Affiliated Hospital of Hebei Medical University and Affiliated Hospital of Hebei University. None of the recruited patients received preoperative radiotherapy or chemotherapy before biopsy. After resection from glioma patients, the tissues were instantly immersed in liquid nitrogen and then stored at -80°C for later use. The Ethics Committee of the Second Affiliated Hospital of Hebei Medical University and Affiliated Hospital of Hebei University authorized this study. Written informed consent was obtained under a grant from every glioma patient. Histopathological examination was independently confirmed by two pathologists.

### Total RNA isolation and quality control

In line with the instructions from the manufacturer, a HiPure Total RNA Mini Kit (Magen) was employed to extract total RNA from tissues. In addition, a Eukaryote Total RNA Nano 6000 assay was used to assess RNA quality and integrity on an Agilent Technologies 2100 Bioanalyzer (Agilent, Santa Clara, CA, USA). RNA integrity numbers (RINs) were considered integrity values for RNA measurements. The results that showed RINs≥7 were kept for further analysis. After isolation, RNA was stored at -80°C for subsequent experiments.

### RNA-seq analysis

To remove rRNA, the total RNA samples were treated with a RiboErase kit (Human/Mouse/Rat) (Kapa Biosystems, Inc., Woburn, MA). Before the RNA-seq libraries were constructed, linear RNAs were eliminated for subsequent treatment using RNase R (Epicenter Technologies, Madison, WI, USA). Briefly, as specified by the manufacturer, an RNA Hyper Prep Kit (Kapa Biosystems, Inc., Woburn, MA) was employed to construct strand-specific RNA-seq libraries. Following fragmentation, the digested RNA samples were applied to synthesize first- and second-strand cDNAs with the assistance of random hexamer primers; dTTP was substituted by dUTP in the second-strand synthesis reaction. Subsequent to cDNA synthesis, end repair was carried out for the double-stranded products, a single ‘A’ base was added, and the cDNA products were ligated to adaptors. With the aid of magnetic particles, cDNA fragments were obtained for subsequent treatment using uracil DNA glycosylase to achieve removal of the second-strand cDNA. The first-strand cDNA was purified and then PCR-amplified, while a 2100 Agilent Bioanalyzer (Agilent, Santa Clara, CA, USA) was applied for quality control of the libraries. RNA-seq was performed with an Illumina HiSeq X Ten (X10) platform (Illumina, San Diego, CA, USA) via standard protocols on a 150-bp paired-end run. The information of relevant patients participating in the RNA-seq is presented in Supplementary Table 4.

### Quantitative real-time PCR (qRT-PCR)

In line with the manufacturer’s instructions, TRIzol reagent (Life Technologies, CA, USA) was employed to separate total RNA from tissues. Electrophoresis on a denaturing agarose gel was employed to assess RNA integrity. In addition, cDNA was acquired with a High-Capacity cDNA Reverse Transcription kit (Geneseed® II First Strand cDNA Synthesis Kit, Guangzhou, China). For qRT-PCR on an ABI 7500 real-time PCR system (Applied Biosystems, Foster City, CA, USA), a SYBR Green kit (Geneseed® qPCR SYBR® Green Master Mix, Guangzhou, China) was employed. For collection of fluorescent signals, the PCR program was operated for 5 minutes at 95°C prior to 40 thermal cycles, each with 10 sec at 95°C and 32 sec at 60°C. Glyceraldehyde phosphate dehydrogenase (GAPDH) (Sangon Biotech, Shanghai, China) was the endogenous control used to normalize circRNA expression. After PCR, the primer specificity was assessed by melt curve analysis. Using the average of the GAPDH-normalized 2^-ΔΔCt^ values, the circRNA expression levels were comparatively quantified through calculation [62]. The circRNA sequences were obtained from circBase (http://www.circbase.org/) [63] using divergent primers spanning the back-splice junction sites rather than convergent primers. A 1% (w/v) agarose gel was applied to separate the amplified PCR products, which were subjected to confirmation by Sanger sequencing.

### Bioinformatics analysis

The functions and related pathways of the host genes (linear counterparts) and the downstream target genes (mRNA) of dysregulated circRNAs were separately investigated via GO (http://www.geneontology.org/) and Reactome pathway analyses (https://reactome.org/). The miRNAs could potentially bind the differentially expressed circRNAs identified with profiling data were predicted using miRanda [64] software coupled with statistical analysis. Four identified circRNAs and their related top miRNAs were drawn using Cytoscape software (version 3.7.1; http://www.cytoscape.org/). The TargetScan (http://www.targetscan.org/) and miRDB database (http://mirdb.org/) were applied to identify the miRNA-mRNA regulatory interactions. Expression and survival curves for selected target miRNAs and mRNAs were analyzed with microRNA microarray data from the Chinese Glioma Genome Atlas (CGGA) database. The expression and relevant data for target genes in patients with GBM and LGG were also examined from the GEPIA (http://gepia.cancer-pku.cn/), ONCOMINE (https://www.oncomine.org/), and cBioPortal (http://www.cbioportal.org/) databases. The cut-off of the ONCOMINE threshold of the p-value and fold change were defined as 0.0001 and 2, respectively. RBPs and ORF data matching with circRNAs were predicted via data from CSCD (http://gb.whu.edu.cn/CSCD/) [65]. The interaction of RBP genes and cancer pathways was analyzed by GSCALite (http://bioinfo.life.hust.edu.cn/web/GSCALite/). The m^6^A levels and m^6^A sites in these circRNAs were predicted via circBank (http://www.circbank.cn/) and SRAMP (http://www.cuilab.cn/sramp). The prediction of coding protein function of identified circRNAs was analyzed and predicted by circRNADb (http://reprod.njmu.edu.cn/circrnadb) [66].

### Statistical analysis

GraphPad Prism 8 (GraphPad Software, Inc., La Jolla, CA, USA) was used for statistical analysis. Comparisons of two variables in the RNA-seq data were performed using the Student’s t-test. Fold change (FC) ≥ 2 or ≤ 0.5 and p < 0.05 were considered statistically significant in the circRNA-seq analysis. To adjust the p-value in RNA-seq analysis, the false discovery rate was determined by calculations. For each circRNA, the expression level is indicated as the fold change determined by applying the 2^-ΔΔCt^ method for qRT-PCR analysis. All data are denoted as the mean ± S.D. of a minimum of three separate experiments. The Mann-Whitney test was used for data comparison. p-values < 0.05 indicate statistical significance.

### Data Availability Statement

The raw data supporting the conclusions of this article will be made available by the authors, without undue reservation.

## Supplementary Material

Supplementary Figures

Supplementary Table 1

Supplementary Table 2

Supplementary Table 3

Supplementary Table 4

## References

[1] Louis DN, Perry A, Reifenberger G, von Deimling A, Figarella-Branger D, Cavenee WK, Ohgaki H, Wiestler OD, Kleihues P, Ellison DW. The 2016 world health organization classification of tumors of the central nervous system: a summary. Acta Neuropathol. 2016; 131:803–20. 10.1007/s00401-016-1545-1 27157931

[2] Jiang Y, He J, Guo Y, Tao H, Pu F, Li Y. Identification of genes related to low-grade glioma progression and prognosis based on integrated transcriptome analysis. J Cell Biochem. 2020; 121:3099–111. 10.1002/jcb.29577 31886582

[3] Chen LL, Yang L. Regulation of circRNA biogenesis. RNA Biol. 2015; 12:381–88. 10.1080/15476286.2015.1020271 25746834 PMC4615371

[4] Qu S, Yang X, Li X, Wang J, Gao Y, Shang R, Sun W, Dou K, Li H. Circular RNA: a new star of noncoding RNAs. Cancer Lett. 2015; 365:141–48. 10.1016/j.canlet.2015.06.003 26052092

[5] Liu J, Zhao K, Huang N, Zhang N. Circular RNAs and human glioma. Cancer Biol Med. 2019; 16:11–23. 10.20892/j.issn.2095-3941.2018.0425 31119043 PMC6528446

[6] Geng X, Jia Y, Zhang Y, Shi L, Li Q, Zang A, Wang H. Circular RNA: biogenesis, degradation, functions and potential roles in mediating resistance to anticarcinogens. Epigenomics. 2020; 12:267–83. 10.2217/epi-2019-0295 31808351

[7] Ding C, Yi X, Wu X, Bu X, Wang D, Wu Z, Zhang G, Gu J, Kang D. Exosome-mediated transfer of circRNA CircNFIX enhances temozolomide resistance in glioma. Cancer Lett. 2020; 479:1–12. 10.1016/j.canlet.2020.03.002 32194140

[8] Zhang S, Liao K, Miao Z, Wang Q, Miao Y, Guo Z, Qiu Y, Chen B, Ren L, Wei Z, Lin Y, Lu X, Qiu Y. CircFOXO3 promotes glioblastoma progression by acting as a competing endogenous RNA for NFAT5. Neuro Oncol. 2019; 21:1284–96. 10.1093/neuonc/noz128 31504797 PMC6784278

[9] Wang R, Zhang S, Chen X, Li N, Li J, Jia R, Pan Y, Liang H. CircNT5E acts as a sponge of miR-422a to promote glioblastoma tumorigenesis. Cancer Res. 2018; 78:4812–25. 10.1158/0008-5472.CAN-18-0532 29967262

[10] Lu Y, Deng X, Xiao G, Zheng X, Ma L, Huang W. Circ_0001730 promotes proliferation and invasion via the miR-326/Wnt7B axis in glioma cells. Epigenomics. 2019; 11:1335–52. 10.2217/epi-2019-0121 31304776

[11] Chen J, Chen T, Zhu Y, Li Y, Zhang Y, Wang Y, Li X, Xie X, Wang J, Huang M, Sun X, Ke Y. circPTN sponges miR-145-5p/miR-330-5p to promote proliferation and stemness in glioma. J Exp Clin Cancer Res. 2019; 38:398. 10.1186/s13046-019-1376-8 31511040 PMC6737709

[12] Luo Z, Rong Z, Zhang J, Zhu Z, Yu Z, Li T, Fu Z, Qiu Z, Huang C. Circular RNA circCCDC9 acts as a miR-6792-3p sponge to suppress the progression of gastric cancer through regulating CAV1 expression. Mol Cancer. 2020; 19:86. 10.1186/s12943-020-01203-8 32386516 PMC7210689

[13] He J, Huang Z, He M, Liao J, Zhang Q, Wang S, Xie L, Ouyang L, Koeffler HP, Yin D, Liu A. Circular RNA MAPK4 (circ-MAPK4) inhibits cell apoptosis via MAPK signaling pathway by sponging miR-125a-3p in gliomas. Mol Cancer. 2020; 19:17. 10.1186/s12943-019-1120-1 31992303 PMC6986105

[14] Han B, Chao J, Yao H. Circular RNA and its mechanisms in disease: from the bench to the clinic. Pharmacol Ther. 2018; 187:31–44. 10.1016/j.pharmthera.2018.01.010 29406246

[15] Zhang M, Huang N, Yang X, Luo J, Yan S, Xiao F, Chen W, Gao X, Zhao K, Zhou H, Li Z, Ming L, Xie B, Zhang N. A novel protein encoded by the circular form of the SHPRH gene suppresses glioma tumorigenesis. Oncogene. 2018; 37:1805–14. 10.1038/s41388-017-0019-9 29343848

[16] Zhang Y, Geng X, Li Q, Xu J, Tan Y, Xiao M, Song J, Liu F, Fang C, Wang H. m6A modification in RNA: biogenesis, functions and roles in gliomas. J Exp Clin Cancer Res. 2020; 39:192. 10.1186/s13046-020-01706-8 32943100 PMC7500025

[17] Li F, Yi Y, Miao Y, Long W, Long T, Chen S, Cheng W, Zou C, Zheng Y, Wu X, Ding J, Zhu K, Chen D, et al. N^6^-methyladenosine modulates nonsense-mediated mRNA decay in human glioblastoma. Cancer Res. 2019; 79:5785–98. 10.1158/0008-5472.CAN-18-2868 31530567 PMC7360104

[18] Yang Y, Fan X, Mao M, Song X, Wu P, Zhang Y, Jin Y, Yang Y, Chen LL, Wang Y, Wong CC, Xiao X, Wang Z. Extensive translation of circular RNAs driven by N^6^-methyladenosine. Cell Res. 2017; 27:626–41. 10.1038/cr.2017.31 28281539 PMC5520850

[19] Tang C, Klukovich R, Peng H, Wang Z, Yu T, Zhang Y, Zheng H, Klungland A, Yan W. ALKBH5-dependent m6A demethylation controls splicing and stability of long 3'-UTR mRNAs in male germ cells. Proc Natl Acad Sci USA. 2018; 115:E325–33. 10.1073/pnas.1717794115 29279410 PMC5777073

[20] Tang C, Xie Y, Yu T, Liu N, Wang Z, Woolsey RJ, Tang Y, Zhang X, Qin W, Zhang Y, Song G, Zheng W, Wang J, et al. m^6^A-dependent biogenesis of circular RNAs in male germ cells. Cell Res. 2020; 30:211–28. 10.1038/s41422-020-0279-8 32047269 PMC7054367

[21] Huang R, Zhang Y, Bai Y, Han B, Ju M, Chen B, Yang L, Wang Y, Zhang H, Zhang H, Xie C, Zhang Z, Yao H. N^6^-methyladenosine modification of fatty acid amide hydrolase messenger RNA in circular RNA STAG1-regulated astrocyte dysfunction and depressive-like behaviors. Biol Psychiatry. 2020; 88:392–404. 10.1016/j.biopsych.2020.02.018 32387133

[22] Zhong S, Wang J, Zhang Q, Xu H, Feng J. CircPrimer: a software for annotating circRNAs and determining the specificity of circRNA primers. BMC Bioinformatics. 2018; 19:292. 10.1186/s12859-018-2304-1 30075703 PMC6090782

[23] Hansen TB, Jensen TI, Clausen BH, Bramsen JB, Finsen B, Damgaard CK, Kjems J. Natural RNA circles function as efficient microRNA sponges. Nature. 2013; 495:384–88. 10.1038/nature11993 23446346

[24] Shan K, Liu C, Liu BH, Chen X, Dong R, Liu X, Zhang YY, Liu B, Zhang SJ, Wang JJ, Zhang SH, Wu JH, Zhao C, Yan B. Circular noncoding RNA HIPK3 mediates retinal vascular dysfunction in diabetes mellitus. Circulation. 2017; 136:1629–42. 10.1161/CIRCULATIONAHA.117.029004 28860123

[25] Zhang Y, Lin X, Geng X, Shi L, Li Q, Liu F, Fang C, Wang H. Advances in circular RNAs and their role in glioma (review). Int J Oncol. 2020; 57:67–79. 10.3892/ijo.2020.5049 32319596 PMC7252450

[26] Xia X, Li X, Li F, Wu X, Zhang M, Zhou H, Huang N, Yang X, Xiao F, Liu D, Yang L, Zhang N. A novel tumor suppressor protein encoded by circular AKT3 RNA inhibits glioblastoma tumorigenicity by competing with active phosphoinositide-dependent Kinase-1. Mol Cancer. 2019; 18:131 10.1186/s12943-019-1056-5 31470874 PMC6716823

[27] Kun-Peng Z, Xiao-Long M, Lei Z, Chun-Lin Z, Jian-Ping H, Tai-Cheng Z. Screening circular RNA related to chemotherapeutic resistance in osteosarcoma by RNA sequencing. Epigenomics. 2018; 10:1327–46. 10.2217/epi-2018-0023 30191736

[28] Song X, Zhang N, Han P, Moon BS, Lai RK, Wang K, Lu W. Circular RNA profile in gliomas revealed by identification tool UROBORUS. Nucleic Acids Res. 2016; 44:e87. 10.1093/nar/gkw075 26873924 PMC4872085

[29] Bachmayr-Heyda A, Reiner AT, Auer K, Sukhbaatar N, Aust S, Bachleitner-Hofmann T, Mesteri I, Grunt TW, Zeillinger R, Pils D. Correlation of circular RNA abundance with proliferation—exemplified with colorectal and ovarian cancer, idiopathic lung fibrosis, and normal human tissues. Sci Rep. 2015; 5:8057. 10.1038/srep08057 25624062 PMC4306919

[30] Zhang PF, Wei CY, Huang XY, Peng R, Yang X, Lu JC, Zhang C, Gao C, Cai JB, Gao PT, Gao DM, Shi GM, Ke AW, Fan J. Circular RNA circTRIM33-12 acts as the sponge of MicroRNA-191 to suppress hepatocellular carcinoma progression. Mol Cancer. 2019; 18:105. 10.1186/s12943-019-1031-1 31153371 PMC6545035

[31] Liu H, Bi J, Dong W, Yang M, Shi J, Jiang N, Lin T, Huang J. Invasion-related circular RNA circFNDC3B inhibits bladder cancer progression through the miR-1178-3p/G3BP2/SRC/FAK axis. Mol Cancer. 2018; 17:161. 10.1186/s12943-018-0908-8 30458784 PMC6245936

[32] Dong W, Bi J, Liu H, Yan D, He Q, Zhou Q, Wang Q, Xie R, Su Y, Yang M, Lin T, Huang J. Circular RNA ACVR2A suppresses bladder cancer cells proliferation and metastasis through miR-626/EYA4 axis. Mol Cancer. 2019; 18:95. 10.1186/s12943-019-1025-z 31101108 PMC6524247

[33] Kong Y, Li Y, Luo Y, Zhu J, Zheng H, Gao B, Guo X, Li Z, Chen R, Chen C. circNFIB1 inhibits lymphangiogenesis and lymphatic metastasis via the miR-486-5p/PIK3R1/VEGF-C axis in pancreatic cancer. Mol Cancer. 2020; 19:82. 10.1186/s12943-020-01205-6 32366257 PMC7197141

[34] Zhao Q, Liu J, Deng H, Ma R, Liao JY, Liang H, Hu J, Li J, Guo Z, Cai J, Xu X, Gao Z, Su S. Targeting mitochondria-located circRNA SCAR alleviates NASH via reducing mROS output. Cell. 2020; 183:76–93.e22. 10.1016/j.cell.2020.08.009 32931733

[35] Han D, Wang Y, Wang Y, Dai X, Zhou T, Chen J, Tao B, Zhang J, Cao F. The tumor-suppressive human circular RNA CircITCH sponges miR-330-5p to ameliorate doxorubicin-induced cardiotoxicity through upregulating SIRT6, survivin, and SERCA2a. Circ Res. 2020; 127:e108–25. 10.1161/CIRCRESAHA.119.316061 32392088

[36] Isik A, Ramanathan R. Approaches to the treatment of pilonidal sinus disease, clinical practice in 2019. Int Wound J. 2020; 17:508–09. 10.1111/iwj.13265 31710171 PMC7948731

[37] Geng X, Lin X, Zhang Y, Li Q, Guo Y, Fang C, Wang H. Exosomal circular RNA sorting mechanisms and their function in promoting or inhibiting cancer. Oncol Lett. 2020; 19:3369–80. 10.3892/ol.2020.11449 32269609 PMC7114721

[38] Wang H, Han M, Whetsell W Jr, Wang J, Rich J, Hallahan D, Han Z. Tax-interacting protein 1 coordinates the spatiotemporal activation of Rho GTPases and regulates the infiltrative growth of human glioblastoma. Oncogene. 2014; 33:1558–69. 10.1038/onc.2013.97 23563176 PMC3965267

[39] Pettee KM, Becker KN, Alberts AS, Reinard KA, Schroeder JL, Eisenmann KM. Targeting the mDia formin-assembled cytoskeleton is an effective anti-invasion strategy in adult high-grade glioma patient-derived neurospheres. Cancers (Basel). 2019; 11:392. 10.3390/cancers11030392 30897774 PMC6468841

[40] Teng H, Wang P, Xue Y, Liu X, Ma J, Cai H, Xi Z, Li Z, Liu Y. Role of HCP5-miR-139-RUNX1 feedback loop in regulating Malignant behavior of glioma cells. Mol Ther. 2016; 24:1806–22. 10.1038/mt.2016.103 27434586 PMC5112034

[41] Zhao K, Cui X, Wang Q, Fang C, Tan Y, Wang Y, Yi K, Yang C, You H, Shang R, Wang J, Kang C. RUNX1 contributes to the mesenchymal subtype of glioblastoma in a TGFβ pathway-dependent manner. Cell Death Dis. 2019; 10:877. 10.1038/s41419-019-2108-x 31754093 PMC6872557

[42] Xu C, Yu Y, Ding F. Microarray analysis of circular RNA expression profiles associated with gemcitabine resistance in pancreatic cancer cells. Oncol Rep. 2018; 40:395–404. 10.3892/or.2018.6450 29781033

[43] Zhang J, Liu H, Hou L, Wang G, Zhang R, Huang Y, Chen X, Zhu J. Circular RNA_LARP4 inhibits cell proliferation and invasion of gastric cancer by sponging miR-424-5p and regulating LATS1 expression. Mol Cancer. 2017; 16:151. 10.1186/s12943-017-0719-3 28893265 PMC5594516

[44] Huang X, Li Z, Zhang Q, Wang W, Li B, Wang L, Xu Z, Zeng A, Zhang X, Zhang X, He Z, Li Q, Sun G, et al. Circular RNA AKT3 upregulates PIK3R1 to enhance cisplatin resistance in gastric cancer via miR-198 suppression. Mol Cancer. 2019; 18:71. 10.1186/s12943-019-0969-3 30927924 PMC6441201

[45] Yu J, Xu QG, Wang ZG, Yang Y, Zhang L, Ma JZ, Sun SH, Yang F, Zhou WP. Circular RNA cSMARCA5 inhibits growth and metastasis in hepatocellular carcinoma. J Hepatol. 2018; 68:1214–27. 10.1016/j.jhep.2018.01.012 29378234

[46] Liang WC, Wong CW, Liang PP, Shi M, Cao Y, Rao ST, Tsui SK, Waye MM, Zhang Q, Fu WM, Zhang JF. Translation of the circular RNA circβ-catenin promotes liver cancer cell growth through activation of the Wnt pathway. Genome Biol. 2019; 20:84. 10.1186/s13059-019-1685-4 31027518 PMC6486691

[47] Han D, Li J, Wang H, Su X, Hou J, Gu Y, Qian C, Lin Y, Liu X, Huang M, Li N, Zhou W, Yu Y, Cao X. Circular RNA circMTO1 acts as the sponge of microRNA-9 to suppress hepatocellular carcinoma progression. Hepatology. 2017; 66:1151–64. 10.1002/hep.29270 28520103

[48] Li Z, Yanfang W, Li J, Jiang P, Peng T, Chen K, Zhao X, Zhang Y, Zhen P, Zhu J, Li X. Tumor-released exosomal circular RNA PDE8A promotes invasive growth via the miR-338/MACC1/MET pathway in pancreatic cancer. Cancer Lett. 2018; 432:237–50. 10.1016/j.canlet.2018.04.035 29709702

[49] Liu F, Zhang J, Qin L, Yang Z, Xiong J, Zhang Y, Li R, Li S, Wang H, Yu B, Zhao W, Wang W, Li Z, Liu J. Circular RNA EIF6 (Hsa_circ_0060060) sponges miR-144-3p to promote the cisplatin-resistance of human thyroid carcinoma cells by autophagy regulation. Aging (Albany NY). 2018; 10:3806–20. 10.18632/aging.101674 30540564 PMC6326687

[50] Wang J, Zhang Q, Zhou S, Xu H, Wang D, Feng J, Zhao J, Zhong S. Circular RNA expression in exosomes derived from breast cancer cells and patients. Epigenomics. 2019; 11:411–21. 10.2217/epi-2018-0111 30785332

[51] Wang F, Yang L, Sun J, Zheng J, Shi L, Zhang G, Cui N. Tumor suppressors microRNA-302d and microRNA-16 inhibit human glioblastoma multiforme by targeting NF-κB and FGF2. Mol Biosyst. 2017; 13:1345–54. 10.1039/c7mb00139h 28497156

[52] Wang H, Zhi H, Ma D, Li T. MiR-217 promoted the proliferation and invasion of glioblastoma by repressing YWHAG. Cytokine. 2017; 92:93–102. 10.1016/j.cyto.2016.12.013 28126486

[53] Du WW, Zhang C, Yang W, Yong T, Awan FM, Yang BB. Identifying and characterizing circRNA-protein interaction. Theranostics. 2017; 7:4183–91. 10.7150/thno.21299 29158818 PMC5695005

[54] Zeng Y, Du WW, Wu Y, Yang Z, Awan FM, Li X, Yang W, Zhang C, Yang Q, Yee A, Chen Y, Yang F, Sun H, et al. A circular RNA binds to and activates AKT phosphorylation and nuclear localization reducing apoptosis and enhancing cardiac repair. Theranostics. 2017; 7:3842–55. 10.7150/thno.19764 29109781 PMC5667408

[55] Zheng X, Huang M, Xing L, Yang R, Wang X, Jiang R, Zhang L, Chen J. The circRNA circSEPT9 mediated by E2F1 and EIF4A3 facilitates the carcinogenesis and development of triple-negative breast cancer. Mol Cancer. 2020; 19:73. 10.1186/s12943-020-01183-9 32264877 PMC7137343

[56] Yang Y, Liu X, Cheng L, Li L, Wei Z, Wang Z, Han G, Wan X, Wang Z, Zhang J, Chen C. Tumor suppressor microRNA-138 suppresses low-grade glioma development and metastasis via regulating IGF2BP2. Onco Targets Ther. 2020; 13:2247–60. 10.2147/OTT.S232795 32214825 PMC7082711

[57] Di Timoteo G, Dattilo D, Centrón-Broco A, Colantoni A, Guarnacci M, Rossi F, Incarnato D, Oliviero S, Fatica A, Morlando M, Bozzoni I. Modulation of circRNA metabolism by m^6^A modification. Cell Rep. 2020; 31:107641. 10.1016/j.celrep.2020.107641 32402287

[58] Chai Y, Xie M. LINC01579 promotes cell proliferation by acting as a ceRNA of miR-139-5p to upregulate EIF4G2 expression in glioblastoma. J Cell Physiol. 2019; 234:23658–66. 10.1002/jcp.28933 31187495

[59] Zhou C, Molinie B, Daneshvar K, Pondick JV, Wang J, Van Wittenberghe N, Xing Y, Giallourakis CC, Mullen AC. Genome-wide maps of m6A circRNAs identify widespread and cell-type-specific methylation patterns that are distinct from mRNAs. Cell Rep. 2017; 20:2262–76. 10.1016/j.celrep.2017.08.027 28854373 PMC5705222

[60] Chen RX, Chen X, Xia LP, Zhang JX, Pan ZZ, Ma XD, Han K, Chen JW, Judde JG, Deas O, Wang F, Ma NF, Guan X, et al. N^6^-methyladenosine modification of circNSUN2 facilitates cytoplasmic export and stabilizes HMGA2 to promote colorectal liver metastasis. Nat Commun. 2019; 10:4695. 10.1038/s41467-019-12651-2 31619685 PMC6795808

[61] Dai F, Wu Y, Lu Y, An C, Zheng X, Dai L, Guo Y, Zhang L, Li H, Xu W, Gao W. Crosstalk between RNA m^6^ A modification and non-coding RNA contributes to cancer growth and progression. Mol Ther Nucleic Acids. 2020; 22:62–71. 10.1016/j.omtn.2020.08.004 32911345 PMC7486578

[62] Livak KJ, Schmittgen TD. Analysis of relative gene expression data using real-time quantitative PCR and the 2(-Delta Delta C(T)) method. Methods. 2001; 25:402–08. 10.1006/meth.2001.1262 11846609

[63] Glažar P, Papavasileiou P, Rajewsky N. circBase: a database for circular RNAs. RNA. 2014; 20:1666–70. 10.1261/rna.043687.113 25234927 PMC4201819

[64] Betel D, Wilson M, Gabow A, Marks DS, Sander C. The microRNA.org resource: targets and expression. Nucleic Acids Res. 2008; 36:D149–53. 10.1093/nar/gkm995 18158296 PMC2238905

[65] Xia S, Feng J, Chen K, Ma Y, Gong J, Cai F, Jin Y, Gao Y, Xia L, Chang H, Wei L, Han L, He C. CSCD: a database for cancer-specific circular RNAs. Nucleic Acids Res. 2018; 46:D925–29. 10.1093/nar/gkx863 29036403 PMC5753219

[66] Chen X, Han P, Zhou T, Guo X, Song X, Li Y. circRNADb: a comprehensive database for human circular RNAs with protein-coding annotations. Sci Rep. 2016; 6:34985. 10.1038/srep34985 27725737 PMC5057092

