# Supplementary material for: Screening and functional prediction of differentially expressed circular RNAs in human glioma of different grades
Source: Aging (Albany NY). 2020 Dec 11;13(2):1989–2014. doi: 10.18632/aging.202192 (PMC7880344; doi:10.18632/aging.202192)
Supplement: Supplementary Figures [file aging-13-202192-s001.pdf]

SUPPLEMENTARY FIGURES

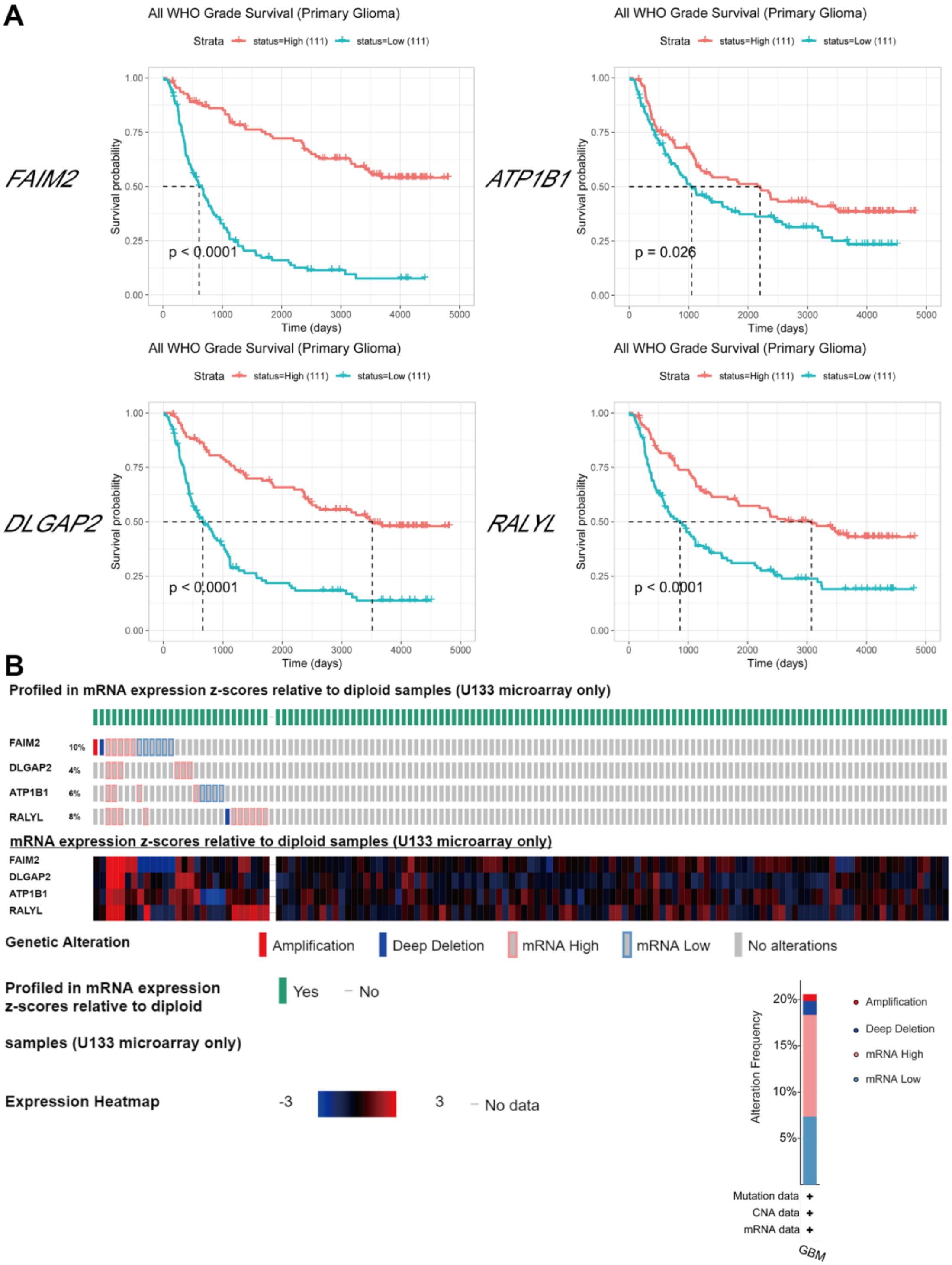

Supplementary Figure 1. Survival analysis and genomic alterations of 4 target mRNAs. (**A**) Survival analysis of 4 target mRNAs by CGGA. (**B**) Summarizes genomic alterations in the 4 queried genes, *FAIM2*, *DLGAP2*, *ATP1B1*, and *RALYL,* across a set of GBM samples by cBioPortal. Each row represents a gene, and each column represents a tumor sample. Red bars represent gene amplifications, and blue bars represent deep deletions.

A

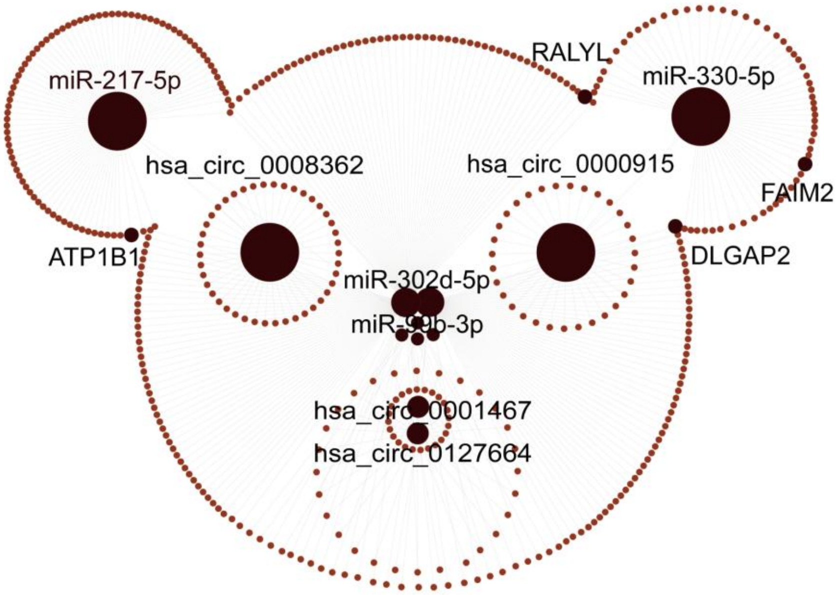

Supplementary Figure 2. CircRNA-miRNA-mRNA network map. (**A**) network predicting target miRNAs of hsa_circ_0000915, hsa_circ_0127664, hsa_circ_0008362, and hsa_circ_0001467 and their top target genes. CircRNAs, target miRNAs and genes are represented by brown nodes. Gray lines indicate a regulatory relationship.

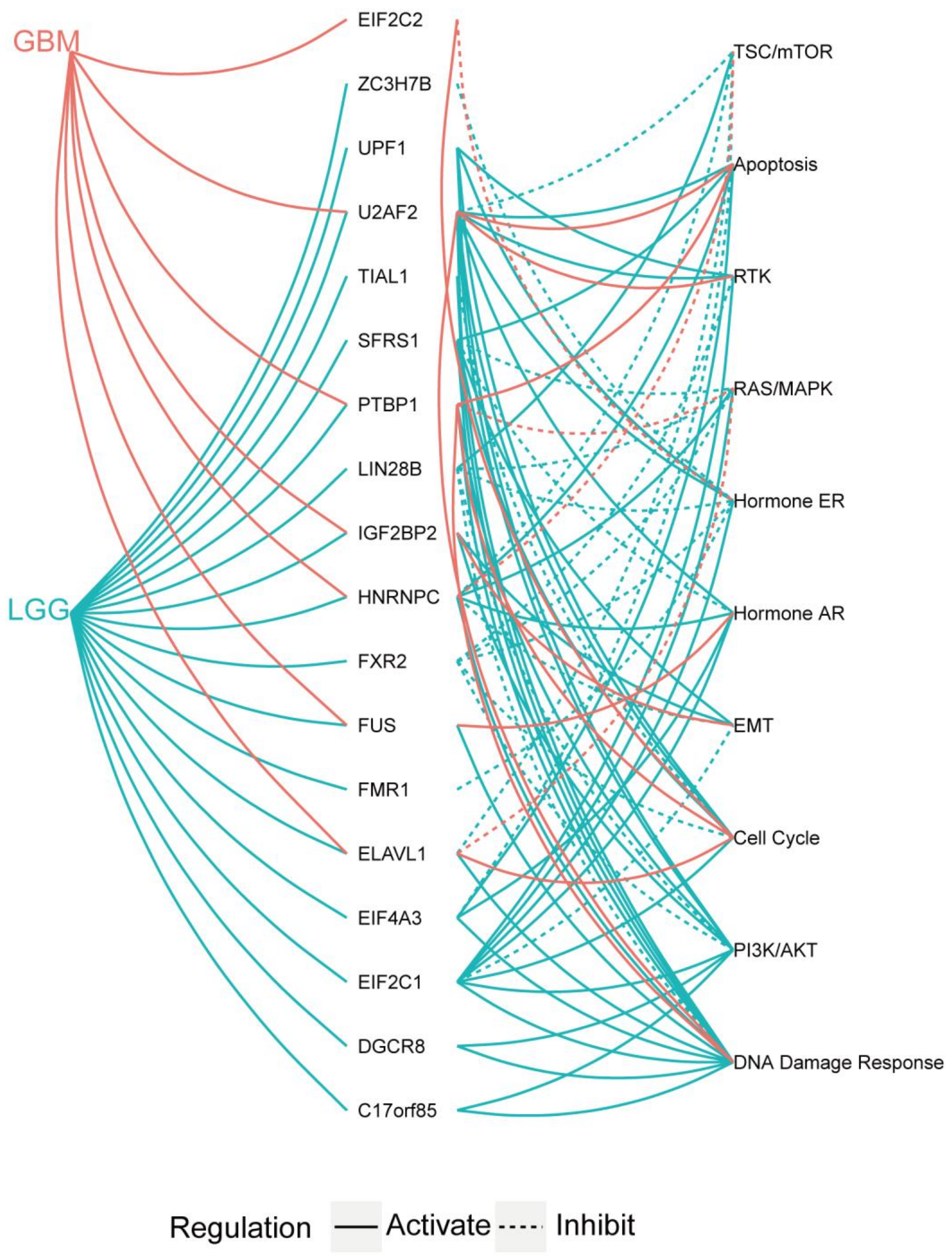

Supplementary Figure 3. Interaction map of RBP genes and activation and inhibition of cancer pathways.

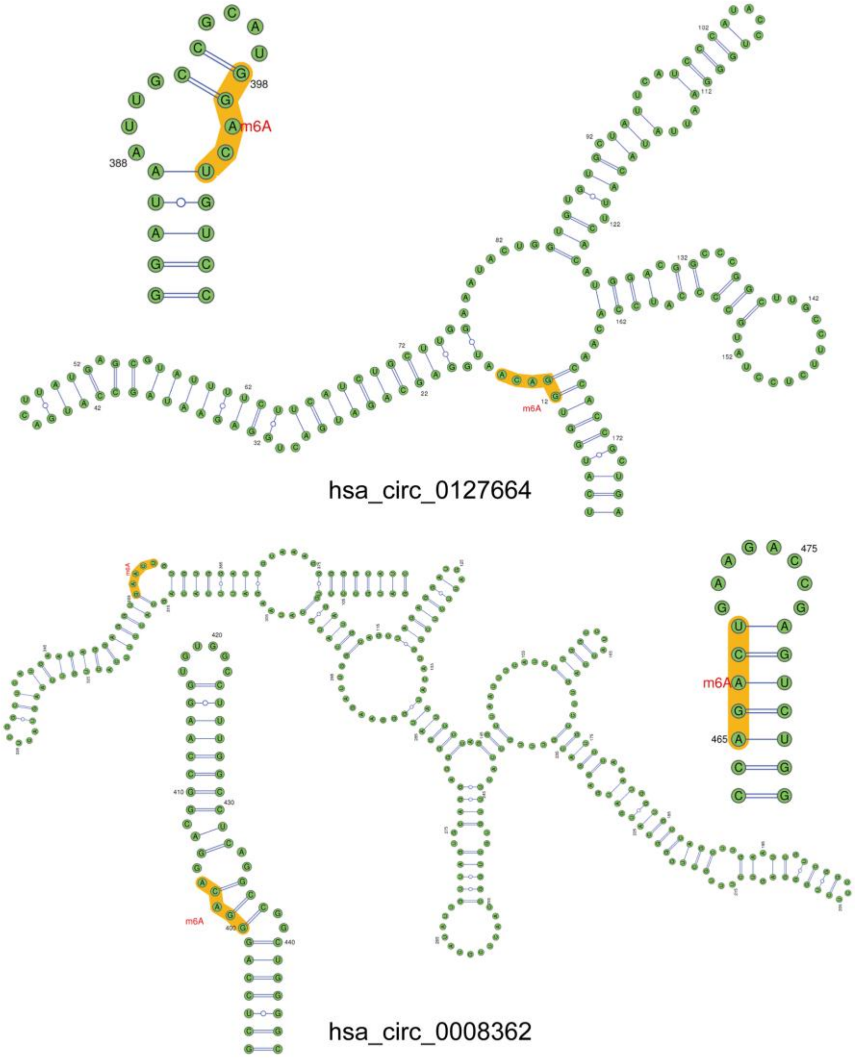

Supplementary Figure 4. Visualization of the local m^{6}A site structure of circRNAs.
