# Supplementary material for: Screening and functional prediction of differentially expressed circular RNAs in human glioma of different grades
Source: Aging (Albany NY). 2020 Dec 11;13(2):1989–2014. doi: 10.18632/aging.202192 (PMC7880344; doi:10.18632/aging.202192)
Supplement: Supplementary Table 1 [file aging-13-202192-s002.pdf]

SUPPLEMENTARY TABLE

Supplementary Table 1. Primer sequences of candidate circRNAs.

Gene	Forward and reverse primer	Product length (bp)
hsa_circ_0127664	hsa_circ_0127664-F1 CCGAGCTTGTGAAAGTTGTT<br>hsa_circ_0127664-R1 GGCCGTCCATGTGAATGTAT	150
hsa_circ_0008362	hsa_circ_0008362-F1 AGCCCATTACTCAGGATGAC<br>hsa_circ_0008362-R1 TCTGACTGCATCTCCACTTC	115
hsa_circ_0000915	hsa_circ_0000915-F1 CGGCCCCCAAGGCAGGGAAC<br>hsa_circ_0000915-R3 CTCCAGCGACGTCTGCAGATGTACG	119
hsa_circ_0001467	hsa_circ_0001467-F2 AGACTTTGATAGTTGCGGTGCTGTT<br>hsa_circ_0001467-R2 ACGGCCAGCAAATACTGCCA	142
GAPDH	GAPDH-F AGAAGGCTGGGGCTCATTTG<br>GAPDH-R GCAGGAGGCATTGCTGATGAT	140
