# Supplementary material for: Screening and functional prediction of differentially expressed circular RNAs in human glioma of different grades
Source: Aging (Albany NY). 2020 Dec 11;13(2):1989–2014. doi: 10.18632/aging.202192 (PMC7880344; doi:10.18632/aging.202192)
Supplement: Supplementary Table 4 [file aging-13-202192-s005.pdf]

SUPPLEMENTARY TABLE

Supplementary Table 4. Clinical information for relevant patients in RNA-seq.

Patient	Age	Gender	WHO grade
High grade1	65	Female	WHO IV
High grade2	50	Male	WHO III
High grade3	53	Female	WHO IV
High grade4	78	Female	WHO IV
High grade5	36	Female	WHO IV
High grade6	53	Female	WHO IV
Low grade1	53	Male	WHO II
Low grade2	64	Male	WHO I-II
Low grade3	37	Female	WHO II
Low grade4	35	Male	WHO II
Low grade5	32	Female	WHO II
Low grade6	38	Male	WHO II
Normal1	65	Female	
Normal2	53	Female	
Normal3	35	Male	
Normal4	38	Male	
Normal5	36	Female	
